# Heterozygous deletion of both sclerostin (*Sost*) and connexin43 (*Gja1*) genes in mice is not sufficient to impair cortical bone modeling

**DOI:** 10.1371/journal.pone.0187980

**Published:** 2017-11-17

**Authors:** Susan K. Grimston, Francesca Fontana, Marcus Watkins, Roberto Civitelli

**Affiliations:** Department of Internal Medicine, Division of Bone and Mineral Diseases, Musculoskeletal Research Center, Washington University School of Medicine, St. Louis, Missouri, United States of America; Augusta University, UNITED STATES

## Abstract

Connexin43 (Cx43) is the main gap junction protein expressed in bone forming cells, where it modulates peak bone mass acquisition and cortical modeling. Genetic ablation of the Cx43 gene (*Gja1*) results in cortical expansion with accentuated periosteal bone formation associated with decreased expression of the Wnt inhibitor sclerostin. To determine whether sclerostin (*Sost)* down-regulation might contribute to periosteal expansion in *Gja1* deficient bones, we took a gene interaction approach and crossed mice harboring germline null alleles for *Gja1* or *Sost* to generate single *Gja1*^*+/–*^and *Sost*^*+/–*^and double *Gja1*^*+/–*^*;Sost*^*+/–*^heterozygous mice. In vivo μCT analysis of cortical bone at age 1 and 3 months confirmed increased thickness in *Sost*^*–/–*^mice, but revealed no cortical abnormalities in single *Gja1*^+/–^or *Sost*^*+/–*^mice. Double heterozygous *Gja1*^*+/–*^*Sost*^*+/–*^also showed no differences in mineral density, cortical thickness, width or geometry relative to wild type control mice. Likewise, 3-point bending measurement of bone strength revealed no significant differences between double *Gja1*^*+/–*^*;Sost*^*+/–*^or single heterozygous and wild type mice. Although these data do not exclude a contribution of reduced sclerostin in the cortical expansion seen in *Gja1* deficient bones, they are not consistent with a strong genetic interaction between *Sost* and *Gja1* dictating cortical modeling.

## Introduction

Bone remodeling and homeostasis depend on the concerted actions of bone forming and resorbing cells, whose function is orchestrated by endocrine and paracrine factors, and by direct cell-cell interactions. Gap junctions are intercellular channels formed by the pairing of two hemichannels, or connexons, on the membrane of opposing cells, allowing direct transfer of small cytoplasmic molecules. Connexin 43 (Cx43) is the main gap junction protein expressed in bone, where it directs peak bone mass acquisition, cortical modeling, and mechano-responsiveness [1–3]. Underscoring its importance for bone development and homeostasis, spontaneous mutations of *GJA1* (Cx43 gene) cause rare skeletal disorders, oculodentodigital dysplasia (ODDD) and recessive craniometaphyseal dysplasia [1, 4]. In mice, conditional deletion of *Gja1* in osteogenic cells results in age-dependent accentuation of periosteal expansion, leading to diaphyseal widening and cortical thinning. This cortical phenotype is the result of enhanced periosteal bone formation and endocortical bone resorption [5, 6]. At the cellular level, Cx43 participates in a number of homeostatic processes, and can regulate signaling through different mechanisms, such as direct cell-cell transfer of second messengers or small metabolites, hemichannel-mediated release of paracrine factors, or by providing a scaffold for intracellular signaling components through its cytoplasmic domain [2, 7].

Interestingly, mice with a germline ablation of the sclerostin gene (*Sost*^*–/–*^), a potent Wnt inhibitor, also have expanded cortices [8]; and we and others have shown that mice with conditional deletion of *Gja1* in osteogenic lineage cells have decreased *Sost* expression in bone [9, 10]. *Gja1*deficient mice display increased sensitivity to mechanical loading, with enhanced periosteal bone formation responses to tibial compression or axial loading of the ulna [10]. On the other hand, cortical bone loss after skeletal unloading is significantly reduced in conditional *Gja1* deficient mice [11]. Notably, *Sost*^*–/–*^mice are also hyper-responsive to anabolic loading regimens in terms of periosteal bone formation, while they are partially protected from bone loss following mechanical unloading [12]. Finally, *Sost* expression decreases upon mechanical unloading and decreases with loading [13–15].

Considering the similar age-related periosteal expansion in *Gja1* and *Sost* deficient mice, the modulatory action of Cx43 and sclerostin on cortical bone response to loading and unloading, and the decreased *Sost* expression in *Gja1* deficient bones [5, 10], we hypothesized that decreased sclerostin production by Cx43-deficient osteocytes might contribute to the accentuated bone formation of *Gja1* deficient mice. Here, we tested this hypothesis using a gene interaction approach, and studied mice with combined *Sost* and *Gja1* haploinsufficiency. Since loss of Cx43 in osteoblasts and osteocytes reduces sclerostin expression [10], we posited that sclerostin production would be further reduced in compound heterozygous mice, thus leading to enhanced Wnt activation and cortical expansion, phenocopying conditional *Gja1* ablation. Our results show that loss of a single *Sost* allele does not alter cortical bone geometry or strength, consistent with previously reported normal total-body bone density in these mice [8]. More to the point, results also show that combined *Sost* and *Gja1* haploinsufficiency has no consistent effect on cortical bone architecture or strength, suggesting that the action of Cx43 on cortical bone modeling is mostly independent of reduced *Sost* expression.

## Materials and methods

### Animals

To generate mice carrying one null allele of *Gja1* and *Sost* (*Gja1*^*–/+*^*;Sost*^*–/+*^) we crossed single heterozygous *Gja1*^*–/+*^ [16] and *Sost*^*–/+*^ [8] mice (mixed background). Wild type littermates from the same crosses, and *Sost* knockout (*Sost*^*–/–*^) mice obtained by crossing male and female *Sost*^*–/+*^ mice were used as controls. All the genotype groups were obtained at the expected Mendelian frequencies, and did not exhibit overt skeletal abnormalities at birth. Genotyping was performed by PCR on genomic DNA extracted from mouse tails using the HotSHOT method, as previously described [5, 9]. Primers to detect wild type and null *Gja1* and *Sost* alleles were as previously described [8, 17]. Mice were fed an ad libitum regular chow (PicoLab Rodent Diet 20, 5053; TestDiet/LabDiet, St. Louis, MO) and housed in cages containing 2–5 animals each, in a room maintained at constant 25°C on a 12-hour light/dark cycle. Mice were euthanized by carbon dioxide inhalation as per Animal Studies Committee recommended protocol. All procedures were approved by the Animal Studies Committee of Washington University (protocol number: 20140279).

### Gene expression analysis

Tibiae were cleaned of all connective tissue, then the proximal epiphysis was cut off, and the bone marrow was removed by centrifugation (10,000 rpm for 10 sec). Tibiae were then flash frozen in liquid nitrogen for storage at −80°C. Bones were mechanically dissociated in Trizol reagent (Thermo Fisher Scientific, Waltham, MA USA) with a Bullet Blender Storm (Midwest Scientific, St Louis, MA, USA) for 2–4 cycles of 5 minutes. RNA was extracted with chloroform then the aqueous phase was purified with RNeasy Mini Kit (Qiagen Valencia, CA, USA) columns according to the manufacturer's directions, including DNAse treatment (Qiagen, Valencia, CA, USA). Reverse transcription using High capacity cDNA reverse transcription kit (Applied Biosystems, Carlsbad, CA, USA) was performed on 0.5μg of RNA, and 10ng of template cDNA were used for qPCR with SYBR Premix Ex Taq (Tli RNase H Plus) (Takara Bio, Shiga Prefecture, Japan) and target-specific primers (sequences in supplementary information), and analyzed with a QuantStudio 3 Real-Time PCR System (Applied Biosystems, Carlsbad, CA, USA). Target gene expression was normalized to 18S using the ΔΔCT method.

### Bone microstructure

For in vivo analysis of bone microstructure and mineralization, animals were subjected to in vivo scanning using μCT (VIVA μCT40, Scanco Medical, AG, Switzerland) using previously described parameters [11]. Microstructural analysis of cortical bone was also as described [3, 11].

### Bone histology and histomorphometric analysis

Bone samples were prepared as previously described [3]. Quantitative histomorphometry was performed using a commercial software (OSTEO II, Bioquant, Nashville, TN, USA), and standard parameters of bone remodeling were determined according to the American Society for Bone and Mineral Research guidelines.

### Bone biomechanics

For assessment of bone strength, dissected femurs were tested in three-point bending to failure or fracture using methods described previously [18]. Briefly, specimens were stabilized over two supports placed 7 mm apart in an Instron 8841 apparatus (Instron, Norwood, MA, USA). A loading force was applied in the anteroposterior direction midway between the two supports by a displacement ramp at a rate of0.03 mm/s. Force and displacement data were collected at100 Hz (Labview 5.0, National Instruments, Austin, TX, USA), and test curves were analyzed as previously described [11, 19].

### Statistics

Statistical analyses were performed using Prism 5 (GraphPad Software, Inc., La Jolla, CA, USA). Group means were analyzed by analysis of variance (ANOVA) followed by post hoc analysis for multiple group comparisons with the level of significance set at *p*<0.05. Unless otherwise specified, data are expressed as mean ± standard deviation. Preliminary analyses were conducted to confirm that gender did not account for variability in the cortical bone data set, and groups were stratified by gender for easier graphic and tabular representation.

## Results

**Fig 1:**
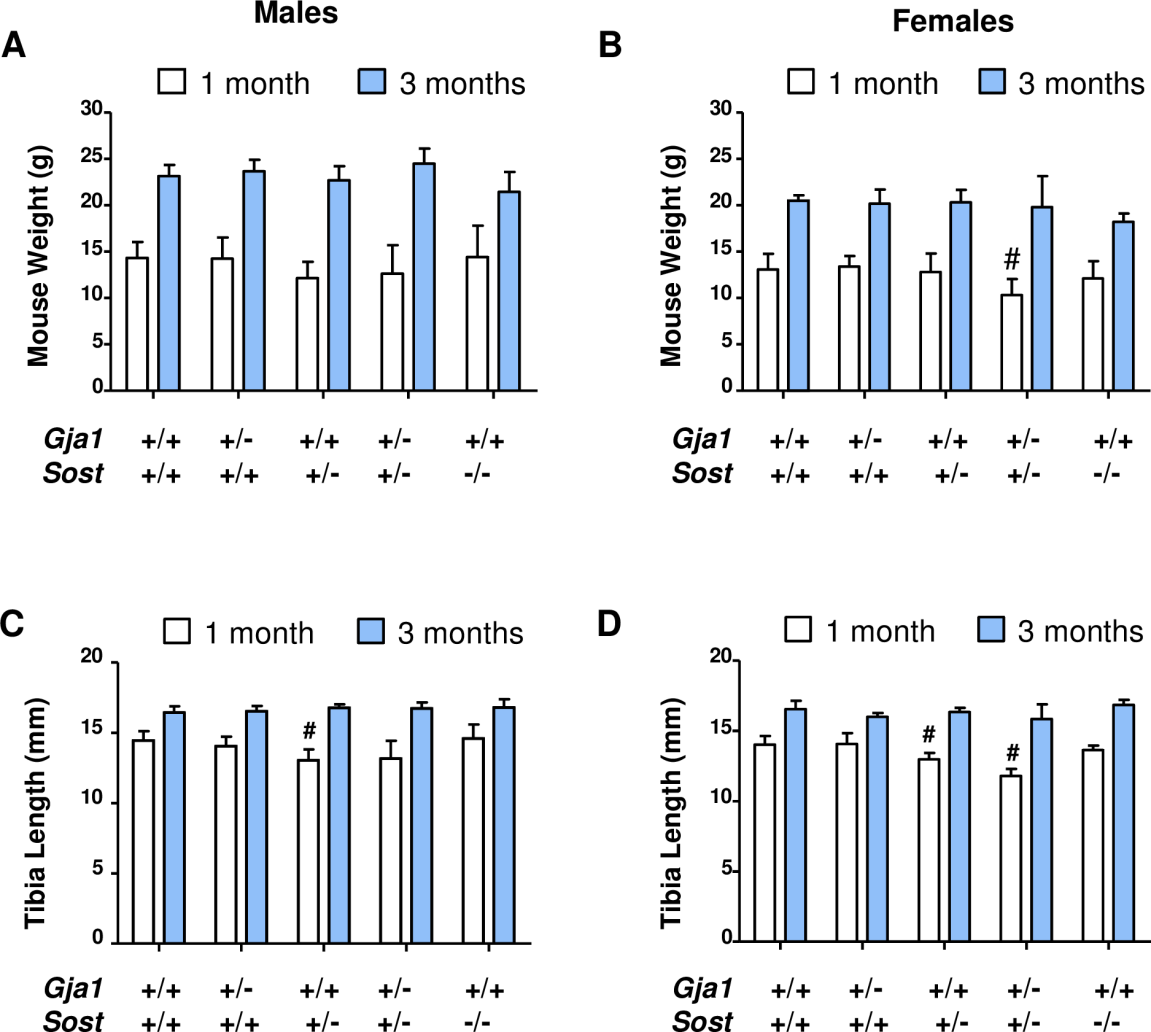
Normal growth in *Gja1*^*–/+*^;*Sost*^*–/+*^ compound heterozygous mice relative to control littermates. (A) Body weight in 1 (open bars) vs. 3 months (solid) old male mice (n = 4–13 per group per time-point) and (B) 1 vs. 3 months old female mice (n = 3–13 per group per time-point); two-way ANOVA p<0.001 for age in both genders, genotype p = 0.016 in females, ns in males (C) tibial length measured by in-vivo CT at 1 vs. 3 months of age in males (n = 4–9 per group per time-point) and (D) in female mice (n = 3–7 per group per time-point); # Dunnett’s post-hoc test p<0.01 relative to WT at one month of age; two-way ANOVA p<0.001 for age in both genders, genotype p<0.01 in females, p = 0.055 in males.

We first monitored growth of the different mutants. At 1 month of age, there was no difference in body weight across genotypes in males (Fig 1A); whereas *Gja1*^*–/+*^;*Sost*^*–/+*^ double heterozygous female mice had 20% lower body weight relative to the other genotypes (Fig 1B). However, this difference was no longer seen at 3 months of age, when body weight was the same across genotypes in both genders (Fig 1A and 1B). Tibia length increased with age, as expected, in all animals, with some variation across genotypes. At one month of age, the tibiae of *Sost*^*–/+*^ mice were significantly shorter than wild type controls in both male and females (about 10% and 8%, respectively); however, this difference was not seen at 3 months (Fig 1C and 1D). Consistent with the reduced body weight, at 1 month the tibiae of double *Gja1*^*-/+*^;*Sost*^*–/+*^ heterozygous female were about 16% shorter than wild type (Fig 1D). By 3 months of age, however, all inter-genotype differences were lost, suggesting that loss of one *Sost* allele, alone or in combination with loss of one *Gja1* allele, does not induce consistent or permanent effects on mouse skeletal growth.

**Fig 2:**
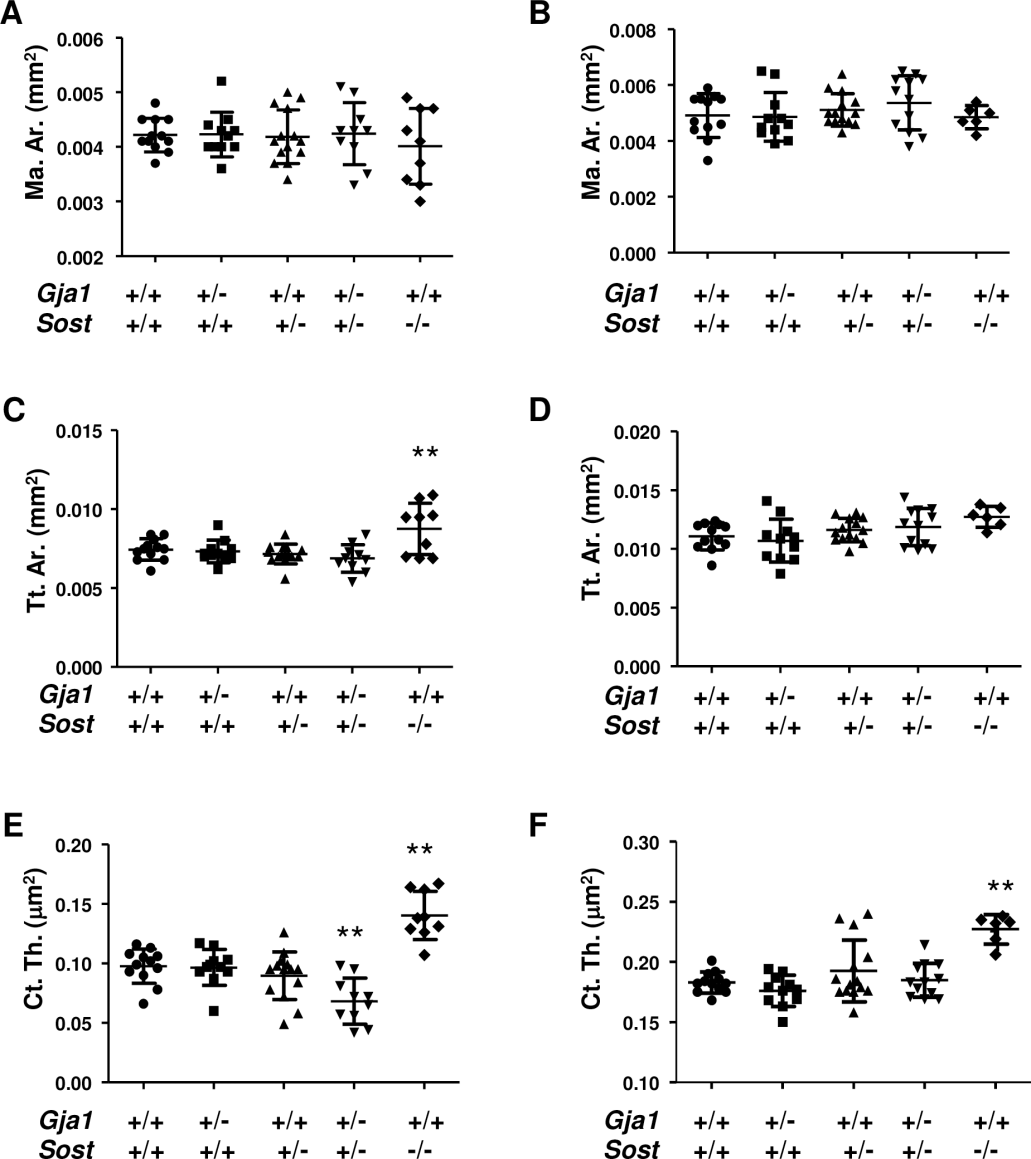
In vivo μCT analysis of cortical bone in *Gja1*^*–/+*^ and *Sost*^*–/+*^ mutant mice. In vivo analysis of cortical bone at the mid-point of tibial diaphysis in 1 (left: A, C, E, G) and 3 month old mice (right: B, D, F, H). (A-B) marrow area; (C-D) Total area; (E-F) Cortical thickness; (G-H) representative images. **p<0.01 relative to WT in Dunnett’s test for multiple comparisons in ANOVA; data from males and females (n = 6–14 per group). See Table 1 for gender-specific analyses (two-way ANOVA for gender ns).

In vivo μCT analysis revealed that at 1 and 3 months of age all cortical morphometric parameters were normal in single *Gja1*^*–/+*^ and *Sost*^*–/+*^ heterozygous null mice. Likewise, in *Sost*^*–/–*^mice we did not detect significant differences in marrow area relative to other genotypes at either one (Fig 2A) or three months (Fig 2B). Total cortical area was higher in *Sost*^*–/–*^mice at age 1 month (about 40%, p<0.001), but such difference was no longer present at 3 months of age (Fig 2D). As anticipated from previous reports [8], cortical thickness was higher in *Sost*^*–/–*^at both age 1 and 3 months (about 25%) (Fig 2E and 2F). Increased cortical thickness was observed in *Sost*^*–/–*^mice of both genders, while a significant expansion of cortical tissue area was seen only in males (Table 1). Intriguingly, cortical thickness in *Gja1*^*–/+*^;*Sost*^*–/+*^ mice was lower than in wild type mice, but this difference disappeared at 3 months of age (Fig 2E and 2F).

**Table 1:** In vivo μCT analysis of tibial diaphysis at the mid-point.

	Sex	Age (mo)		WT	*Gja1*^*–/+*^	*Sost*^*–/+*^	*Gja1*^*–/+*^; *Sost*^*–/+*^	*Sost*^*–/–*^	*P* (ANOVA)
Marrow area (Mar. Ar.) mm^2^	M	1	Mean	0.0042800	0.0044400	0.0042380	0.0046000	0.0043400	0.699
			SD	0.0002049	0.0004615	0.0004658	0.0004123	0.0006542	
		3	Mean	0.0053800	0.0054600	0.0051670	0.0059830	0.0048750	0.092
			SD	0.0005263	0.0009555	0.0006185	0.0005845	0.0002062	
	F	1	Mean	0.0041710	0.0040500	0.0041170	0.0038800	0.0036000	0.312
			SD	0.0003729	0.0002811	0.0005565	0.0004817	0.0005477	
		3	Mean	0.0045710	0.0043670	0.0050200	0.0047500	0.0048000	0.639
			SD	0.0007868	0.0003830	0.0005718	0.0009182	0.0008485	
Tissue Area (Tt. Ar.) mm^2^	M	1	Mean	0.0075000	0.0075200	0.0070880	0.0074200	0.0095000**	0.003
			SD	0.0004848	0.0008585	0.0007717	0.0007662	0.0015940	
		3	Mean	0.0053800	0.0054600	0.0051670	0.0059830	0.0048750	0.199
			SD	0.0005263	0.0009555	0.0006185	0.0005845	0.0002062	
	F	1	Mean	0.0074140	0.0071670	0.0072670	0.0063400	0.0078250	0.070
			SD	0.0008275	0.0005955	0.0003724	0.0006309	0.0012500	
		3	Mean	0.0106000	0.0097170	0.0112800	0.0108500	0.0121500	0.1259
			SD	0.0012580	0.0012020	0.0011900	0.0011950	0.0010610	
Cortical Thickness (Ct. Th.) μm^2^	M	1	Mean	0.0964000	0.0920000	0.0845000	0.0776000	0.1458000**	0.001
			SD	0.0135000	0.0204700	0.0199400	0.0225200	0.0266000	
		3	Mean	0.1842000	0.1786000	0.1983000	0.1888000	0.228000*	0.017
			SD	0.0124200	0.0106900	0.0305200	0.0167500	0.0148100	
	F	1	Mean	0.0985700	0.1005000	0.0965000	0.058800**	0.1335000**	<0.001
			SD	0.0160800	0.0091160	0.0198900	0.0105200	0.0061370	
		3	Mean	0.1820000	0.1738000	0.1824000	0.1808000	0.2255000**	<0.001
			SD	0.0059720	0.0153300	0.0084440	0.0106800	0.0091920	
Sample number	M	1	N	5	5	8	5	5	
		3	N	5	5	9	6	4	
	F	1	N	7	6	6	5	4	
		3	N	7	6	5	6	2	

Analysis of in vivo μCT, showing mean, standard deviation (SD) and number of subjects analyzed (N) for male (M) and female (F) mice aged 1 or 3 months. Analysis of variance (ANOVA: *p* value in the last column); followed by Dunnet’s post-hoc test for multiple comparisons (*p<0.05 and **p<0.01 relative to WT).

**Fig 3:**
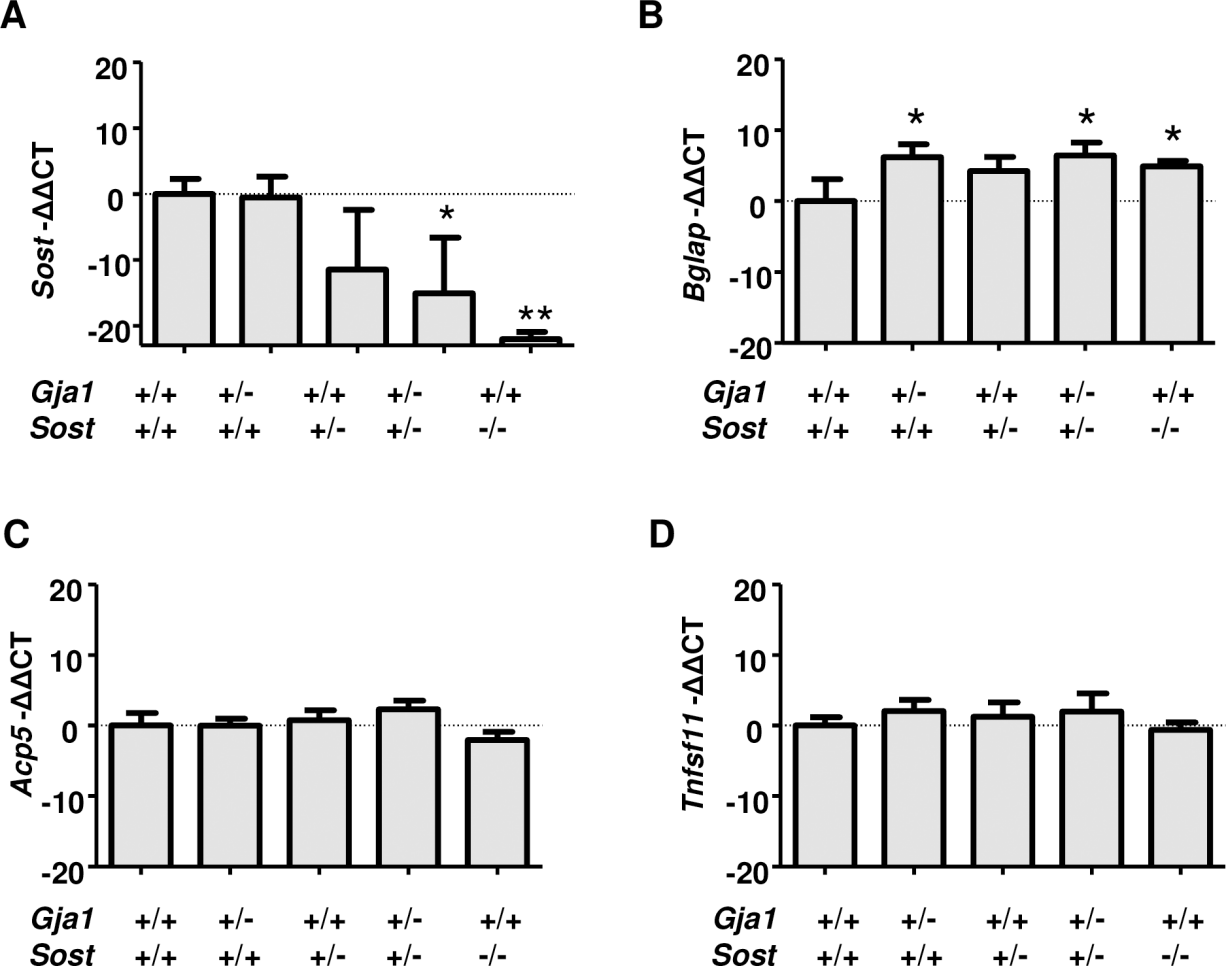
Gene expression in cortical bones of *Gja1*^*–/+*^ and *Sost*^*–/+*^ mutant mice. Abundance of mRNA for sclerostin (*Sost*) (A), osteocalcin (*Bglap*) (B), TRAP5b (*Acp5*) (C), and RANKL (*Tnfsf11*) (D) extracted from the tibial diaphysis and assessed by RT-qPCR. Average and standard deviation of biological triplicates normalized to WT. * p<0.05 and **p<0.01 relative to WT in Dunnett’s test for multiple comparisons after ANOVA.

To validate our model and determine how combined heterozygous loss of *Gja1* and *Sost* affects expression of genes involved in osteoclastogenesis and bone formation we analyzed mRNA from cortical bone extracts of adult mice by RT-qPCR. As expected, *Sost* mRNA was almost undetectable in *Sost*^*–/–*^mice, and it was decreased, though not significantly, in *Sost*^*-/+*^ bones. Importantly, *Sost* mRNA was significantly reduced relative to WT controls in *Gja1*^*-/+*^*; Sost*^*-/+*^ mice (Fig 3A). Furthermore, we noted a small but significant increase in osteocalcin (*Bglap*) mRNA in *Gja1*^*-/+*^, *Sost*^*—+*^ and *Gja1*^*-/+*^*; Sost*^*-/+*^ mice relative to controls, By contrast, neither the osteoclast marker tartrate-resistant acid phosphatase 5b (TRAP5b, *Acp5*) (Fig 3C), or the pro-osteoclastogenic factor receptor activator of nuclear factor kappa-B ligand (RANKL, *Tnfsf11*) (Fig 3D) were altered in any of the mutant mice.

We had previously shown that conditional *Gja1* ablation in osteogenic cells leads to increased periosteal formation and cortical thinning by enhancing endocortical bone resorption. To determine whether osteoclast number is altered in our mutant mice, we performed tartrate-resistant acid phosphatase (TRAP) staining and hystomorphometric analysis of paraffin embedded sections of tibiae. To capture an osteoclast-rich area, we analyzed the proximal metaphysis of 1 month-old tibiae. Consistent with lack of major structural abnormalities on cortical bone and unaltered whole-tissue gene expression, we found that 1-month old *Gja1*^*–/+*^*;Sost*^*–/+*^ mice had a normal number of TRAP-positive, multinucleated cells (Table 2). Osteoblast surface was increased in *Sost*^*–/+*^ but not in *Sost*^*–/–*^(or *Gja1*^*–/+*^) mice, and there were no differences among genotypes in osteoblast number (Table 2), suggesting the high osteoblast surface in *Sost*^*–/+*^ may reflect a random finding.

**Table 2:** Static cellular histomorphometric parameters in *Gja1*^*–/+*^ and *Sost*^*–/+*^ mutant mice.

		WT	*Gja1*^*–/+*^	*Sost*^*–/+*^	*Gja1*^*–/+*^*;Sost*^*–/+*^	*Sost*^*–/–*^
Oc.S/BS	Mean	0.11840	0.15690	0.19230	0.10830	0.13410
	SD	0.02887	0.02914	0.05067	0.04861	0.10150
N. Oc/BS	Mean	5.22400	7.05600	3.29300	4.32800	3.60200
	SD	0.38800	2.28700	0.96150	1.64500	1.04200
Ob. S/BS	Mean	0.05057	0.06397	0.18290*	0.04302	0.08462
	SD	0.01691	0.01888	0.07381	0.01480	0.03814
N. Ob/BS	Mean	5.23700	5.31600	7.04900	5.73500	6.90400
	SD	1.40800	2.36100	2.82700	0.88760	2.41700

Histomorphometry analysis on TRAP-stained sections from 1 month old male mice (n = 3-5/group).

*p<0.05 relative to WT controls by Dunnet’s post-hoc test for multiple comparisons.

**Fig 4:**
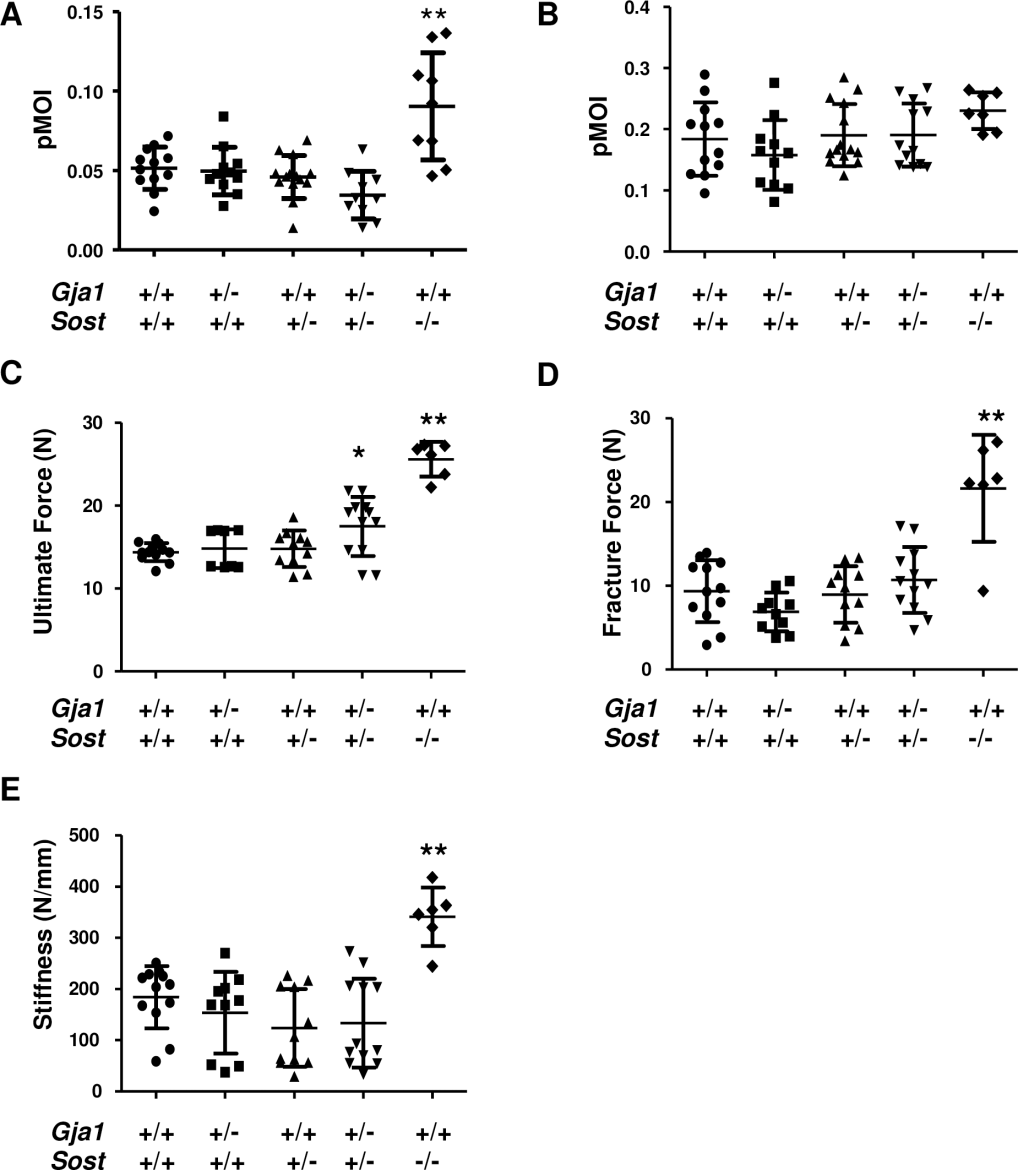
Biomechanical properties in *Gja1*^*–/+*^ and *Sost*^*–/+*^ mutant mice. Polar moment of inertia (pMOI) at 1 (A) and 3 (B) months of age measured by μCT analysis of the tibia as in Fig 2 (n = 7–14 per group). Three-point bending biomechanical testing in femurs of 3 month old mice, reporting ultimate force (C), fracture force (D), and stiffness (E) (n = 6–12 per group). * p<0.05 and **p<0.01 relative to WT in Dunnett’s test for multiple comparisons in ANOVA.

We then asked whether single or compound heterozygous deletion would affect bone strength, which is contributed to by material properties in addition to bone architecture. Consistent with the normal cortical parameters, the polar moment of inertia (pMOI), a geometric index of bone strength, was not different between each single or compound *Gja1*^*–/+*^;*Sost*^*–/+*^ mutants and wild type mice. A transient increase was noted in *Sost*^*–/–*^mice at age 1 month, although the difference was lost at 3 months (Fig 4A and 4B). Direct biomechanical testing of bone strength was performed by 3-point bending on tibiae from 3 month old mice. Consistent with the geometric data, bone strength (ultimate force, fracture force and stiffness) was not different between single *Gja1*^*–/+*^ and *Sost*^*–/+*^ heterozygous and wild type mice (Fig 4C and 4E). However, the compound *Gja1*^*–/+*^;*Sost*^*–/+*^ mutants exhibited a 20% higher ultimate force relative to wild-type controls, but no significant differences in fracture force or stiffness (Fig 4C and 4E). As expected from previous reports, all the 3 biomechanical parameters were significantly higher in *Sost*^*–/–*^mice (Fig 4C and 4E).

## Discussion

This genetic interaction study demonstrates that combined haploinsufficiency of *Gja1* and *Sost* does not recapitulate the cortical bone expansion that occurs with ablation of either gene, despite significant reduction in sclerostin expression. While not fully excluding participation of reduced sclerostin expression in the pathogenesis of periosteal expansion in *Gja1* deficiency, these results indicate that other mechanisms are involved in Cx43 modulation of cortical bone modeling.

Previous work from our and other groups has shown that mice with conditional ablation of *Gja1* in cells of the osteogenic lineage have decreased *Sost* expression [9, 10]. These mice also share similarities in the response to mechanical loading with *Sost* null mice; specifically, they show enhanced periosteal bone apposition in response to mechanical loading [9, 20]. We tested the hypothesis that reduced sclerostin production observed in Cx43 deficient bones might contribute to their accentuated periosteal expansion. We anticipated that combined loss of one allele of both *Gja1* and *Sost* would lead to further reduction of sclerostin relative to each single heterozygous null mutant, resulting in cortical expansion. Contrary to such expectations, our results demonstrate that compound *Gja1*^*–/+*^*;Sost*^*–/+*^ mutants have a normal cortex at 3 months of age, despite reduced levels of sclerostin. The lower cortical thickness (and the tendentially lower pMOI) observed in these mutants at 1 month of age is apparently inconsistent with the higher ultimate force evident at 3 months of age; however, since the morphological features normalize by 3 months of age, the thinner cortices at 1 month may simply reflect a modest developmental delay. On the other hand, we observed the expected increased cortical thickness in *Sost*^*–/–*^mice at 1 and 3 months [8]. This is likely the result of reduced endocortical expansion and accentuated periosteal apposition, changes that are below the detection limit of histomorphometry. Therefore, while not excluding that sclerostin may participate in the cortical expansion of Cx43-deficient mice, our study demonstrates that ablation of a single *Sost* allele is not sufficient to cause the cortical phenotype in a *Gja1* heterozygous null background.

Heterozygous loss of *Gja1* in vivo causes detectable abnormalities of cardiac conductance [21]. In the skeleton, we previously reported that calvaria cells from *Gja1*^*–/+*^ mice have reduced Cx43 protein levels, decreased cell coupling, and reduced mineralization, thus exhibiting an in vitro hypomorph phenotype relative to *Gja1* knockout [17]. Despite these in vitro abnormalities, here we confirm that heterozygous *Gja1* loss does not result in an overt skeletal phenotype [22], implying that a single copy of *Gja1* is sufficient for skeletal development and homeostasis, at least in normal homeostatic conditions. We have previously reported that conditional ablation of *Gja1* in the osteogenic lineage induces cortical thinning and marrow area expansion through increased osteoclastogenesis. Indeed, blockade of osteoclast activity with bisphosphonates rescues cortical thinning [3]. Furthermore, Cx43-deficient osteogenic cells produce lower abundance of the bone resorption inhibitor, osteoprotegerin (*Tnfrsf11b*) [5], and higher levels of the bone resorption stimulator, RANKL (*Tnfsf11*) [6], resulting in enhanced osteoclast differentiation [5]. Here, we show that a single *Gja1* allele in osteoblastic cells is sufficient to support normal osteoclast numbers *in vivo* and a normal cortical morphology. Intriguingly, we find increased *Bgalp* expression in single *Gja1*^*+/–*^, and compound *Gja1*^*+/–*^*;Sost*^*+/–*^mutants, as well as in *Sost* null bones, suggesting that both genes participate in the regulation of *Bgalp*. However, there is no synergistic effect in the compound mutants, further stressing lack of genetic interactions. Notably, these results are consistent with increased circulating levels of ostocalcin (*Bgalp* gene product) both in osteoblast-specific *Gja1* ablated mice [5] and in *Sost* null mice [8]. However, since osteoblast number, total bone area and cortical thickness are unaltered in *Gja1*^*+/–*^and *Gja1*^*+/–*^*;Sost*^*+/–*^mice, the increased expression of a marker of osteoblast function in these *Gja1* haploinsufficient mice may reflect only modest changes in osteoblast function, not sufficient to alter bone architecture, at least within 3 months of life. It is also worth noting that Cx43 is positively regulated by canonical Wnt/β-catenin signaling [23], adding to the complexity in the cross-regulation between *Sost* and *Gja1*.

We show that loss of one *Sost* allele results in decreased sclerostin production, implying constitutively increased Wnt signaling and osteoblast activity. Yet, we see no structural or biomechanical abnormalities in heterozygous *Sost*^*+/–*^mice. Wnt signaling stimulation is followed by a number of compensatory effects and negative feedback loops, that eventually turn off the signal [24]. Studies on rodents [25] and non-human primates [26] have shown that while administration of neutralizing anti-sclerostin antibodies rapidly uncouples bone turnover favoring bone formation, the remodeling cycle eventually re-couples despite continuous treatment; this is associated with increased circulating levels of sclerostin and Dkk-1, another Wnt inhibitor. While complete loss of sclerostin might not be fully compensated, thus resulting in high bone mass in mice and humans, partial loss of sclerostin as it occurs in heterozygous *Sost*^*+/–*^mice might be overridden by homeostatic mechanisms, thus preventing the development of a skeletal phenotype.

Our data demonstrate that combined heterozygous deletion of *Sost* and *Gja1* do not induce the cortical expansion we anticipated based upon our genetic interaction model. Therefore, while not excluding a role of sclerostin, our results demonstrate that the cortical bone modeling abnormalities present in mice with conditional ablation of *Gja1* in bone forming cells is not mediated by decreased sclerostin abundance only.

## Supporting information

S1 Table Primer sequences. (XLSX)

S2 Table Supporting data on mouse growth. (XLSX)

S3 Table Supporting data on bone microstructure. (XLSX)

S4 Table Supporting data on gene expression. (XLSX)

S5 Table Supporting data on histomorphometry. (XLSX)

S6 Table Supporting data on biomechanics. (XLSX)
